# Investigating the interplay between gaming disorder and functional impairments in professional esports gaming

**DOI:** 10.1038/s41598-024-56358-x

**Published:** 2024-03-19

**Authors:** Halley M. Pontes, Hans-Jürgen Rumpf, Špela Selak, Christian Montag

**Affiliations:** 1 School of Psychological Sciences, Birkbeck, University of London, London, UK; 2 Department of Psychiatry and Psychotherapy, University of Lübeck, Lübeck, Germany; 3 National Institute of Public Health, Ljubljana, Slovenia; 4 Department of Molecular Psychology, Institute of Psychology and Education, Ulm University, Helmholtzstr. 8/1, 89081 Ulm, Germany

**Keywords:** Gaming Disorder, World Health Organization, Functional impairments, Esports, Professional gaming, Psychology, Human behaviour

## Abstract

The relationship between Gaming Disorder (GD) and the experience of functional impairments has received considerable theoretical attention in the recent past and current diagnostic approaches underscore the centrality of functional impairments as a requirement for GD diagnosis. However, there is limited empirical evidence illuminating the interplay between GD and functional impairments, particularly among specific vulnerable groups. The present study seeks to bridge this gap by investigating an English-speaking sample (*N* = 5198) comprising an age- and gender-matched group of Professional Gamers (PG, *n* = 2599) and Non-Professional Gamers (NPG, *n* = 2599) sub-sampled from a larger sample of 192,260 individuals. The results revealed that PG were at a greater risk for GD compared to NPG as the prevalence rate of GD among PG (3.31%) was significantly higher and almost doubled that of NPG (1.73%), with PG further exhibiting higher overall GD symptom-load and weekly time spent gaming compared to NPG. Furthermore, PG reported experiencing significantly higher frequency of gaming-related functional impairments compared to NPG, with the in particular affected areas for both PG and NPG being ‘*school and/or work*’, ‘*physical health*’, and ‘*family*’, with other key differences emerging in relation to other outcomes. Overall, the present findings show that not only GD symptom-load but also some functional impairment is higher in PG compared to NPG which highlights the need to develop and support prevention and intervention strategies for this at-risk population.

## Introduction

Recent developments in psychiatric diagnosis and nosology led to the formal recognition of Gaming Disorder (GD) as a *bona fide* mental health disorder and behavioral addiction by the World Health Organization (WHO) in the 11th revision of the *International Classification of Diseases* (ICD-11)^1^. Within the WHO diagnostic framework, GD refers to a problematic pattern of gaming manifested by the following criteria: (i) impaired control over gaming, (ii) greater priority given to gaming to the detriment of competing interests and functional daily activities, (iii) and maintenance and/or increase of gaming regardless its negative consequences. Although these criteria can be endorsed within a 12-month time-frame, when diagnosing GD, these symptoms must be of sufficient severity to result in significant functional impairments across different domains of life, such as personal, family, social, educational, and/or other areas of functioning^1^.

While gaming may present with risks, when engaged judiciously, the activity in itself may be largely harmless, providing a large number of individuals with a pastime activity that may lead to a number of benefits, including but not limited to improving social problem-solving skills in children^2^, physical activity among players of augmented reality games^3^, as well as improving the well-being of players during the pandemic^4^, by specifically mitigating stress, anxiety, depression, and loneliness levels during the period of global lockdown restrictions. A recent study by Matias et al^5^. in a sample of 235 gamers concluded that despite being highly engaged and showing poor sleep quality, most gamers displayed behaviors consistent with a healthy lifestyle.

However, despite its many positive effects, excessive gaming may lead to GD and its accompanying detrimental effects on psychological health and well-being, in terms of increased depression^6^ and levels of problematic online behaviors^7^, hyperactivity and sleep disturbances^8^, digital eye strain^9^, academic performance^10^, and general psychological distress^11^. Relatedly, in the context of the pandemic, individuals played for longer hours, leading to increased time spent playing across different demographics^12,13^, and negative outcomes as shown by previous research^14^, including but not limited to increased anxiety severity^15^, impaired cardiovascular health behavior^16^, reduced well-being^17^,

Within the existing literature, the role of functional impairments in GD has been considered at a theoretical level see ^18^, notably in the context of over-diagnosis of normative gaming behaviors as pathological^19^. However, empirical investigations aiming to disentangle the role of functional impairments in GD remains scant, particularly in relation to the most recent diagnostic framework for GD proposed by the WHO. Recently, Montag and Pontes^20^ have empirically examined the association between self-reported functional impairments due to gaming in individuals potentially experiencing GD among gamers, not belonging to a specific group. The study found that the areas mostly affected by gaming-related functional impairments were school and/or work, followed by psychological health, and family-related functional impairments. Conversely, the least affected area related to friends^20^.

Despite this initial effort by Montag and Pontes^20^, a gap remains in the literature due to the need to further investigate the interplay between GD and functional impairments, particularly in relation to specific groups that may be at increased risk for GD and its negative outcomes. In the context of this broader issue, recent studies examining the relationship between GD and professional players provided useful insights on how GD and related comorbidities may be experienced by specific groups such as Professional Gamers (PG) and Non-Professional Gamers (NPG).

In a recent study by Maldonado-Murciano, et al.^21^, the authors reported that PG showed significantly higher vulnerability to GD compared to NPG, with professional gaming behaviors being strongly associated with higher time spent gaming and greater prevalence rates of GD. In terms of gaming motives, the study by Montag et al^22^. found that specific gaming motives as measured by the Motives for Online Gaming Questionnaire (MOGQ)^23^ were linked to GD symptoms within the WHO framework. More specifically, while PG and those intending to become PG did not differ significantly in their gaming-related motivational patterns, they differed substantially from NPG in relation to social, escapism, competition, coping, skill development, and fantasy motives, but not in the recreational motive.

Similar results have been found with respect to higher scores of esports gamers on social, competition, and skill development gaming motivations compared to recreational gamers^24^. Furthermore, escapism served as a predictor for problematic gaming in this study examining both PG and NPG. A large number of previous studies have used approaches such as the 5th edition of the *Diagnostic and Statistical Manual of Mental Disorders* (DSM-5) to define disordered gaming. Given the debate on overpathologization tendencies within this approach, and the arguable lack of clinical utility^25^ it is important to take functional impairment as additional criterion into account and central factor in GD—as suggested in the diagnostic guidelines of GD in ICD-11.

## The current study

Given the increased relevance and significant growth of esports and professional gaming globally, coupled with the need to further understand the interplay between GD and functional impairments, the present study aims to fill in an existing gap in the literature concerning the experience of GD and specific gaming-related functional impairments among PG. This is key to advance our current understanding of this particular issue and to help ground clinical and diagnostic practices related to GD into empirical findings. It is envisaged that the present study will also contribute to existing debates surrounding the role and implications of functional impairments in GD and that it will also yield empirical findings capable of informing current clinical and diagnostic practices related to GD, particularly in light of competitive and non-competitive gaming activities and individuals.

## Method

### Participants and procedures

An initial sample comprising 192,260 participants was recruited between January 2019 to February 2022 via an online survey platform developed by the researchers (https://www.do-i-play-too-much.com) that was advertised across several media channels and by ESL Gaming, a large esports company (https://about.eslgaming.com/brands/smart-gaming), who provided no direct or indirect financial support nor influenced the study in any way. Participants visiting the online survey platform were invited to partake in the study voluntarily. The study was conducted according to the guidelines of the Declaration of Helsinki and it has received ethical approval by the research team’s University Ethics Committee at Nottingham Trent University (PONTES 2018/95) and all participants were granted anonymity and confidentiality. While those aged between 12 and 15 years were required to provide an electronic parental consent, participants aged at least 16 years provided an electronic informed consent to partake in the study. Note that this study is a follow-up investigation of a previous reports stemming from the same project, with a different research focus ((hence ^20^ and ^21^) see for further details).

After cleaning the data set (see Supplementary Information), a final sample of 5198 English-speaking gamers was obtained by sub-sampling an age- and gender-matched group of PG (*n* = 2599) and NPG (*n* = 2599) using propensity score matching with the Nearest Neighbor Matching method (see^26^). The distinction between professional and non-professional gamers was provided on grounds of the following question: ‘*Are you a professional gamer (i.e., making a living playing video games)?* (yes/no)’. Across both groups of gamers, the observed mean age was 21.29 years (SD = 6.66) with the majority being male (*n* = 4832; 92.96%) and about 33.05% (*n* = 1718) being in a romantic relationship. In terms of academic achievement, nearly half of the sample (i.e., 40.07%; *n* = 2083) reported being a high school graduate.

### Measures

#### Gaming Disorder

The Gaming Disorder Test (GDT)^27^ was utilized to assess disordered gaming symptoms in the past 12 months according to the WHO framework^1^ through four items answered on a five-point Likert scale (1 ‘*never*’—5 ‘*very often*’). Total scores can be obtained by summing up participants’ answers to all items, with greater scores suggesting higher levels of disordered gaming. In line with previous research e.g.,^28,29^, answering all four items with 4 ‘*Often*’ or 5 ‘*Very often*’ was operationalized as being indicative of disordered gaming. A study by Karhulahti and colleagues reported that the GDT had the highest levels of content validity compared to similar measures^30^ while a recent study reported that the GDT showed adequate levels of criterion validity and convergent validity^31^. In the present study, the GDT exhibited adequate levels of reliability (α = 0.74; ω = 0.74; Composite Reliability [CR] = 0.74). For further supporting background information regarding the GDT's psychometric properties and different cultural adaptations, please see: https://www.halleypontes.com/tests/gdt

#### Functional impairments

In order to assess experiences of gaming-related functional impairments, participants were first asked the following question: ‘*Have you experienced a significant problem in your life due to your gaming activity in the last 12 months?*’, those answering ‘*yes*’ to this question were then asked to specify the main area negatively affected. These specific areas included: ‘*family*’; ‘*friends*’; ‘*partner*’; ‘*school and/or work*’; ‘*physical health*’; and ‘*psychological health*’. These specific types of functional impairments were proposed on the basis of a previous research attesting their relevance and role in disordered gaming (see^32–35^).

#### Psychiatric distress

The Kessler Psychological Distress Scale (K6)^36^ is a brief six-item tool yielding a global measure of distress in the past month. All six items are rated on a five-point Likert scale (1 ‘*All of the time*’—5 ‘*None of the time*’), with elevated total scores indicating greater levels of psychiatric distress. Previous research on the psychometric properties of the K6 reported high levels of concurrent validity, incremental validity^37^, and convergent validity^38^. In the present study, the K6 exhibited adequate levels of reliability (α = 0.85; ω = 0.86; CR = 0.85).

#### Loneliness

The three-item Revised Loneliness Scale (R-UCLA)^39^ was used to assess participants’ overall levels of loneliness. All three items were rated on a four-point Likert scale (1 ‘*Never*’—4 ‘*Often*’). Total scores are obtained by summing participants’ responses to all items and higher scores suggest greater levels of loneliness. Previous research found support for the scale’s psychometric validity in terms of criterion validity and strict measurement invariance^40^. In the present study, the R-UCLA showed adequate levels of reliability (α = 0.83; ω = 0.83; CR = 0.83).

#### Attention problems

The Attention Problem Scale^41^ includes three items measuring attentional difficulties. Responses to all three items are given on a five-point Likert scale (1 ‘*Strongly disagree*’—5 ‘*Strongly agree*’) and higher total scores are indicative of greater levels of attention problems. This measure has been used in similar research on gamers, with several studies attesting its use and suitability^42,43^. In the present study, the Attention Problem Scale showed adequate levels of reliability (α = 0.71; ω = 0.75; CR = 0.75).

#### Headache frequency

The Headache Screening Questionnaire^44^ is a 10-item standardized tool for measuring the experience of headache symptoms. In the present study, we utilized the three items assessing the frequency of headaches, which were rated on a three-point Likert scale with higher total scores being indicative of higher frequency of headache experiences. In the original study, the authors reported that the measure presented with high levels of content validity and criterion validity^44^. In the present study, the three items of the Headache Screening Questionnaire had acceptable levels of reliability (α = 0.64; ω = 0.65; CR = 0.65).

#### Insomnia severity

The Insomnia Severity Index^45^ is a brief seven-item tool assessing the severity of insomnia symptoms on a five-point Likert scale (0 ‘*Not at all noticeable*’—4 ‘*Very much noticeable*’) and higher total scores refer to more severe levels of insomnia symptoms. Previous research reported that the measure showed with concurrent validity, predictive validity, and content validity^45^. In the present study, the Insomnia Severity Index had satisfactory levels of reliability (α = 0.80; ω = 0.81; CR = 0.80).

### Statistical analysis and data management

Prior to the main statistical analyses, the data set was cleaned through several steps as previously reported and outlined in another study conducted by the research team see Pontes et al^29^., in addition to including additional cleaning steps due to the unique context of the present study. This approach led to the exclusion of participants that: (i) completed the survey during the COVID-19 pandemic in the years of 2020, 2021, and 2022 (9674 cases removed), (ii) specified being both a PG and intending to become one (246 cases removed), (iii) did not provide informed consent (11,364 cases removed), (iv) were under 12 years old (25,597 cases removed), (v) were over 80 years old (171 cases removed), (vi) did not play any video games in the past 12 months (629 cases removed), (vii) indicated playing a fictitious game in the last 12 months (4646 cases removed), (viii) reported spending 0 h of weekly gaming (428 cases removed), (ix) reported spending more than 119 h of weekly gaming (400 cases removed), (x) reported spending more than 48 h of gaming in the weekend alone (863 cases removed), and (xi) were not proficient in English language (15,149 cases removed). As a result of implementing the aforementioned cleaning strategy, the final sample of PG and NPG included in the analyses was extracted from the sample of 123,093 gamers achieved after employing the data cleaning strategy above.

The following research question and hypotheses will be investigated in the present study: what is the nature and patterns of self-reported functional impairments experienced by professional and non-professional gamers? Based on the existing literature, it is hypothesized that compared to non-professional gamers, professional gamers will report higher risk for GD (hypothesis 1) and greater incidence of functional impairment (hypothesis 2). Furthermore, it is also expected that the nature of the experience in functional impairments will also differ between the two groups when comparing the most commonly experienced functional impairments (hypothesis 3).

All statistical analyses were performed using R v.4.2.2^46^. Specifically, these included conducting (i) descriptive analysis of the main variables; group comparisons with (ii) Chi-squared contingency table tests and goodness-of-fit tests for categorical variables and (iii) Welch’s *t*-test statistics for continuous variables alongside Crammer’s *V* and Cohen’s *d* effect sizes; and (iv) correlational analysis of the main study variables with Holm’s adjusted *p*-values. The following R packages were used to conduct the required analyses: MatchIt v.4.5.0^26^, lavaan v.06-13^47^, semTools v.0.5-6^48^, effsize v.0.8.1^49^.

## Results

### Descriptive analysis of gaming-related behaviors

As can be seen in Table 1, prevalence rates of GD among PG (*n* = 86; 3.31%) was significantly higher and almost double the prevalence rate of GD in NPG (*n* = 45; 1.73%), thus supporting hypothesis 1. Similarly, the severity of GD symptoms was significantly higher among PG (Mean = 9.72; SD = 3.52) compared to NPG (Mean = 8.79; SD = 3.08). Unsurprisingly, PG spent significantly more time gaming a week (Mean = 31.62; SD = 21.52) compared to NPG (Mean = 22.95; SD = 16.21).

**Table 1:** Experience of problems due to gaming among Professional Gamers (PG) and Non-Professional Gamers (NPG).

Variable	PG *n* = 2599	NPG *n* = 2599	Group differences
Problem due to gaming (*n*, %)			*Χ*^2^ (1, 5198) = 55.89, *p* < .001, *V* = .10
Yes	437 (16.81)	254 (9.77)	
No	2,162 (83.19)	2,345 (90.23)	
Area of life affected *(n*, *%)*^a^			
Family	80 (18.31)	30 (11.81)	*χ*^2^(1, 691) = 5.06, *p* = .02, *V* = .08
Friends	51 (11.67)	12 (4.72)	*χ*^2^(1, 691) = 9.35, *p* < .01, *V* = .11
Partner	48 (10.98)	20 (7.87)	*χ*^2^(1, 691) = 1.75, *p* = .19, *V* = .04
School and/or work	144 (32.95)	134 (52.76)	*χ*^2^(1, 691) = 26.20, *p* < .01, *V* = .19
Physical health	60 (13.73)	36 (14.17)	*χ*^2^(1, 691) = 0.03, *p* = .87, *V* = .002
Psychological health	54 (12.36)	22 (8.66)	*χ*^2^(1, 691) = 2.24, *p* = .13, *V* = .05
GD Prevalence (*n*, %)	86 (3.31)	45 (1.73)	*χ*^2^(1, 5198) = 13.16, *p* < .001, *V* = .04
GD Severity (mean, SD)	9.72 (3.52)	8.79 (3.08)	Welch’s *t*(5107.6) = − 10.20, *p* < .01, *d* = .28
Weekly Hours Spent Gaming (mean, SD)	31.62 (21.52)	22.95 (16.21)	Welch’s *t*(4827.6) = − 16.39, *p* < .01, *d* = .45
Psychiatric Distress (mean, SD)	13.50 (5.64)	12.66 (5.08)	Welch’s *t*(5141.4) = − 5.61, *p* < .01, *d* = .16
Loneliness (mean, SD)	6.17 (2.70)	6.20 (2.68)	Welch’s *t*(5195.9) = 0.48, *p* = .632, *d* = .01
Attention Problems (mean, SD)	6.40 (2.78)	6.40 (2.63)	Welch’s *t*(5180.7) = 0.01, *p* = 1, *d* = < .00
Headache Severity (mean, SD)	5.09 (NA)	5.16 (NA)	Welch’s *t*(1888) = 0.95, *p* = .34, *d* = NA
Insomnia Severity (mean, SD)	9.03 (NA)	8.25 (NA)	Welch’s *t*(1909) = − 2.97, *p* < .01, *d* = NA

^a^ Percentages might not add up to 100% due to rounding inaccuracies. NA = Not computed by the software due to missing values.

In regard to the experience of gaming-related problems in the last 12 months, PG reported experiencing significantly more problems compared to NPG. Specifically, approximately one in six PG reported experiencing a problem due to gaming (*n* = 437; 16.81%) compared to nearly one in ten NPG (*n* = 254; 9.77%), providing support to hypothesis 2.

### Experiences of gaming-related functional impairments

In relation to how PG and NPG were affected by the experience of gaming-related functional impairments across all specific areas of life investigated, overall PG exhibited greater levels of functional impairments compared to NPG in relation to ‘*family*', and ‘*friends*’, but with ‘*school and/or work*’ showing an opposing pattern regarding percent. However, PG and NPG did not differ significantly in relation to ‘*partner*’, ‘*physical health*’, and ‘*psychological health*’ functional impairments. This finding does not completely support hypothesis 3.

While the single most predominant area of life affected by gaming-related functional impairments reported by both PG and NPG was ‘*school and/work*’, the second most prevalent for PG was ‘*family*’ (*n* = 80; 18.31%) while for NPG it was ‘*physical health*’ (*n* = 36; 14.17%). Interestingly, the third most predominant area of life affected among PG was ‘*physical health*’ (*n* = 60; 13.73%) while for NPG it was ‘*family*’ (*n* = 30; 11.81%). Finally, in relation to the other variables examined, PG experienced higher levels of functional impairments compared to NPG only in relation to overall psychiatric distress and severity of insomnia with no meaningful differences emerging in loneliness, attention problems, and severity of headache experiences (see Table 1).

### Correlational analysis of the main study variables

To further understand how the experience of gaming-related functional impairments may differ between PG and NPG, a correlational analysis was conducted (see Tables 2 and 3). As for GD severity among PG, the top three strongest associations emerged in relation to psychiatric distress (*r* = 0.31; *p* ≤ 0.001), attention problems (*r* = 0.29; *p* ≤ 0.001), and loneliness (*r* = 0.26; *p* ≤ 0.001). In terms of the six specific areas of life investigated, GD severity was mostly associated with ‘*physical health*’ impairment (*r* = − 0.21; *p* ≤ 0.01). Regarding the associations between specific impairments and the other variables examined, ‘*psychological health*’ showed the strongest association with psychiatric distress (*r* = 0.34; *p* ≤ 0.001).

**Table 2:** Correlational Analysis for Professional Gamers (PG).

Variables	1	2	3	4	5	6	7	8	9	10	11	12
GD Severity (1)	1	.31***	.26***	.29***	.09	.19*	− .12	.13	.07	.07	− .21**	.09
Psychiatric distress (2)		1	.55***	.17*	.34***	.36***	− .16	.09	.07	− .05	− .21**	.34***
Loneliness (3)			1	.11	.10	.30***	− .04	.18*	− .08	− .14	− .03	.21**
Attention problems (4)				1	.04	.30***	− .02	.01	− .19*	.15	− .09	.06
Headache severity (5)					1	.28***	− .18*	.05	.22**	− .14	.00	.16*
Insomnia severity (6)						1	− .11	.05	.04	.00	− .09	.14
Family impairment (7)							1	− .15	− .17*	− .37***	− .20*	− .19*
Friends impairment (8)								1	− .10	− .22**	− .12	− .11
Partner impairment (9)									1	− .26***	− .14	− .13
SAW impairment (10)										1	− .30***	− .28***
PHY impairment (11)											1	− .15
PSY impairment (12)												1

* *p* ≤ .05; ** *p* ≤ .01; *** *p* ≤ .001.

*GD* Gaming Disorder, *SAW* School and/or Work, *PHY* Physical Health, *PSY* Psychological Health.

**Table 3:** Correlational analysis for non-professional gamers (NPG).

Variables	1	2	3	4	5	6	7	8	9	10	11	12
GD severity (1)	1	.44***	.36***	.32***	.02	.39***	− .13	− .05	− .06	.03	.00	.18
Psychiatric distress (2)		1	.52***	.31***	.20*	.41***	− .17	.01	− .18*	.09	.07	.09
Loneliness (3)			1	.22*	.11	.24**	− .07	− .11	− .05	.06	.09	− .02
Attention problems (4)				1	.19*	.29***	− .17	− .25**	− .23**	.19*	− .03	.26**
Headache severity (5)					1	.20*	− .08	− .05	.06	− .10	.10	.12
Insomnia severity (6)						1	− .15	− .01	− .08	− .07	.15	.18*
Family impairment (7)							1	− .07	− .09	− .37***	− .13	− .10
Friends impairment (8)								1	− .06	− .24**	− .09	− .07
Partner impairment (9)									1	− .31***	− .11	− .08
SAW impairment (10)										1	− .46***	− .35***
PHY impairment (11)											1	− .12
PSY impairment (12)												1

* *p* ≤ .05; ** *p* ≤ .01; *** *p* ≤ .001.

*GD* Gaming Disorder, *SAW* School and/or Work, *PHY* Physical Health, *PSY* Psychological Health.

The same analysis for NPG revealed that, GD severity was mostly associated with psychiatric distress (*r* = 0.44; *p* ≤ 0.001), insomnia severity (*r* = 0.39; *p* ≤ 0.001), and loneliness (*r* = 0.36; *p* ≤ 0.001). As for the six specific areas of life investigated, GD severity was not significantly associated with any specific area. However, in terms of the associations between specific impairments and the other variables examined, ‘*psychological health*’ showed the strongest association with attention problems (*r* = 0.26; *p* ≤ 0.01).

### Discussion

The present study investigated the interplay between GD and gaming-related functional impairments in the context of professional and non-professional gaming. To achieve this, an age- and gender-matched sample of PG and NPG was recruited from a larger sample of English-speaking gamers and the results obtained provided important and novel insights on how GD may be related to specific gaming-related functional impairments and how these affect specific areas of life.

The findings obtained suggested that PG were disproportionately affected by GD compared to NPG due to higher prevalence rates. Additionally, the findings highlighted that PG were at much greater risk for GD compared to NPG due to increased incidence and experience of disordered gaming symptoms and higher levels of gaming engagement assessed via weekly time spent gaming. These findings echo the results reported in recent research examining the relationships between GD in esports and PG^21,22^. Since individuals engaged in professional gaming are at a greater risk for GD, it is paramount that further research and preventative initiatives are developed to mitigate the increased dangers of GD to this particular group of individuals who rely on gaming for a living. As previously recommended by, industry-led player protection measures targeting the general population may also be extended to specific groups that are more vulnerable to experiencing GD.

Going beyond the findings of previous studies, the results of this study also suggested that when compared to NPG, PG experienced higher levels of gaming-related functional impairments across relatively similar areas of life, particularly in relation to functional impairments pertaining to social relationships with family and friends, but interestingly with an opposing patterns for school/work (we relate to the percentages). Furthermore, PG and NPG did not differ substantially in terms of functional impairments related to romantic relationships, physical and psychological health as they were similarly affected. Interestingly, the top three areas of life affected by gaming-related functional impairments between PG and NPG were highly comparable, with ‘*school and/or work*’, ‘*physical health*’, and ‘*family*’ being the most prominent areas of life affected.

The correlational findings also suggested that the experience of gaming-related functional impairments among PG and NPG was somewhat slightly distinct. Specifically, among PG, the severity of GD was mostly associated with psychiatric distress, attention problems, and loneliness. Similarly, among NPG, the severity of GD was mostly associated with psychiatric distress, insomnia severity, and loneliness. The differences in levels of psychiatric distress between PG and NPG highlight individual differences concerning how each group experience and cope with distress. All in all, these findings underscore how the experience and severity of GD among both PG and NPG may affect specific indicators of health and well-being, further supporting previous research reporting the detrimental effects stemming from GD^50,51^.

The findings reported in this study present with several important implications for clinical practice and current research. First, from a clinical standpoint the results obtained highly support the consideration and analysis of functional impairments when assessing and diagnosing GD as the experience of functional impairments is critical to determining the extent of problems caused by GD. Thus, the findings encountered lend support to current diagnostic practices in terms of considering functional impairments in GD assessment. Second, clinicians diagnosing GD should pay particular attention to specific areas of life potentially affected by GD to aid targeted treatment efforts as this will likely increase clients’ overall quality of life. In the realm of competitive esports gaming it is worth noting that intrinsic player characteristics such as gameplay self-efficacy may play an important in detrimental health outcomes, including but not limited to competition anxiety^52^.

From a research perspective, future studies are needed to further assess how other functional impairments may be related to GD, beyond the six areas we investigated as a holistic assessment is needed. Finally, it is recommended that future research investigate additional groups, presenting different degrees of vulnerability and exposure to gaming as the experience of gaming-related functional impairments is likely to differ depending on the individuals’ characteristics and individual differences.

Despite the many contributions offered, the present study is not without potential limitations. Although the findings will provide relevant empirical evidence on the relationship between GD and functional impairments, it is worth noting that they bear no direct clinical validity as the sample examined was not clinical (e.g., individuals who underwent treatment, treatment-seekers, etc). Furthermore, despite the fact that this study investigated a large sample of English-speaking gamers, the sampling technique utilized was not random and as such, the findings reported cannot be generalized to all demographics. It is also important to critically consider the validity of the types of functional impairments investigated in the present study as they do not translate to all possible negative experiences disordered gamers may encounter when dealing with GD.

Of direct importance to the present investigation, it is worth noting that researchers have recently proposed a definition for esports players^53^, which includes the consideration of the following three characteristics: being a professional player, being part of an organized team and having experience in competitions, which signifies that the findings reported may have limited validity to individuals exclusively engaged in esports. Finally, despite the rigorous statistical approach adopted, self-reported methodologies may lead to well-known biases, including but not limited to memory recall bias and social desirability. Future research can provide additional meaningful findings by refining their sampling strategy to better screen esports players based on the suggested definition. It is also important that additional related data (e.g., number of tournaments won, team size, etc.) is collected to improve the current understanding of the relationship between GD and esports.

The findings of this study support the public health concerns associated with professional gaming and may be of value to research and practice on esports^54^. Therefore, the presented findings should be considered in light of the increasing need for special care for gamers who aspire to become professional gamers. Collectively, the gaming industry and esports tournament organizers have a responsibility to work collaboratively with researchers and health professionals to promote independent research that is able to inform the development of public health measures capable of preventing the development of GD and able to provide help to affected individuals when GD does occur.

### Supplementary Information

Supplementary Information.

## Data Availability

The dataset used in the current study can be made available from the corresponding author upon reasonable request.
