# Supplementary material for: Investigating the interplay between gaming disorder and functional impairments in professional esports gaming
Source: Sci Rep. 2024 Mar 19;14:6557. doi: 10.1038/s41598-024-56358-x (PMC10951285; doi:10.1038/s41598-024-56358-x)
Supplement: Supplementary file 1 — Supplementary Information. [file 41598_2024_56358_MOESM1_ESM.docx]

**Supplementary Information**

The data cleaning steps employed led to the exclusion of a total of 69,167 participants from the broader sample recruited (*N* = 192,260). The final sample analyzed in the study was subsampled from the clean data set, which included a total of 123,093 participants. The data cleaning approach utilized excluded participants who: filled out the survey during peak pandemic time (i.e., 2020, 2021, and 2022, *n* = 9,674), reported being a professional gamer and intending to become one (*n* = 246), did not provide informed consent (*n* = 11,364), were under the age of 12 years (*n* = 25,597) or over the age of 80 years (*n* = 171), did not play video games in the last 12 months (*n* = 629), reported playing a fictitious video game (*n* = 4,646), reported not playing at least one hour a week (*n* = 428), reported playing more than 119 hours a week (*n* = 400), reported playing more than 48 hours on weekends alone (*n* = 863), reported not being fluent in English (*n* = 15,149).
